# The genital tract and rectal microbiomes: their role in HIV susceptibility and prevention in women

**DOI:** 10.1002/jia2.25300

**Published:** 2019-05-29

**Authors:** Salim S Abdool Karim, Cheryl Baxter, Jo‐Ann S Passmore, Lyle R McKinnon, Brent L Williams

**Affiliations:** ^1^ Centre for the AIDS Programme of Research in South Africa (CAPRISA) University of KwaZulu‐Natal Durban South Africa; ^2^ Department of Epidemiology Columbia University New York NY USA; ^3^ National Health Laboratory Service Cape Town South Africa; ^4^ Institute of Infectious Diseases and Molecular Medicine (IDM) University of Cape Town Cape Town South Africa; ^5^ Department of Medical Microbiology and Infectious Diseases University of Manitoba Winnipeg Manitoba Canada; ^6^ Department of Medical Microbiology University of Nairobi Nairobi Kenya; ^7^ Department of Pathology and Cell Biology Columbia University New York NY USA

**Keywords:** HIV prevention, women, vaginal microbiome, rectal microbiome, genital inflammation, tenofovir gel

## Abstract

**Introduction:**

Young women in sub‐Saharan Africa are disproportionately affected by HIV, accounting for 25% of all new infections in 2017. Several behavioural and biological factors are known to impact a young woman's vulnerability for acquiring HIV. One key, but lesser understood, biological factor impacting vulnerability is the vaginal microbiome. This review describes the vaginal microbiome and examines its alterations, its influence on HIV acquisition as well as the efficacy of HIV prevention technologies, the role of the rectal microbiome in HIV acquisition, advances in technologies to study the microbiome and some future research directions.

**Discussion:**

Although the composition of each woman's vaginal microbiome is unique, a microbiome dominated by *Lactobacillus* species is generally associated with a “healthy” vagina. Disturbances in the vaginal microbiota, characterized by a shift from a low‐diversity, *Lactobacillus*‐dominant state to a high‐diversity non‐*Lactobacillus*‐dominant state, have been shown to be associated with a range of adverse reproductive health outcomes, including increasing the risk of genital inflammation and HIV acquisition. *Gardnerella vaginalis* and *Prevotella bivia* have been shown to contribute to both HIV risk and genital inflammation. In addition to impacting HIV risk, the composition of the vaginal microbiome affects the vaginal concentrations of some antiretroviral drugs, particularly those administered intravaginally, and thereby their efficacy as pre‐exposure prophylaxis (PrEP) for HIV prevention. Although the role of rectal microbiota in HIV acquisition in women is less well understood, the composition of this compartment's microbiome, particularly the presence of species of bacteria from the *Prevotellaceae* family likely contribute to HIV acquisition. Advances in technologies have facilitated the study of the genital microbiome's structure and function. While next‐generation sequencing advanced knowledge of the diversity and complexity of the vaginal microbiome, the emerging field of metaproteomics, which provides important information on vaginal bacterial community structure, diversity and function, is further shedding light on functionality of the vaginal microbiome and its relationship with bacterial vaginosis (BV), as well as antiretroviral PrEP efficacy.

**Conclusions:**

A better understanding of the composition, structure and function of the microbiome is needed to identify opportunities to alter the vaginal microbiome and prevent BV and reduce the risk of HIV acquisition.

**Abbreviations**
BV: bacterial vaginosis
Cpn60: chaperonin‐60
CST: community state type
HEPN: highly exposed seronegative
HIV: human immunodeficiency virus
HMP: Human Microbiome Project
HPV: human papillomavirus
IL: interleukin
IP: inducible protein
LPS: lipopolysaccharide
MCP: monocyte chemotractant protein
MIP: macrophage inflammatory protein
MSM: men who have sex with men
NEC: necrotizing endocolitis
PrEP: pre‐exposure prophylaxis
PRR: prevalence risk ratio
SIV: simian immunodeficiency virus
SMS: Shotgun metagenomic sequencing
STI: sexually transmitted infections
TLR: toll‐like receptor

## 1 Introduction

With 37 million people living with human immunodeficiency virus (HIV) and 1.8 million new infections in 2017, HIV remains a significant global public health problem. Sub‐Saharan Africa bears a disproportionate HIV burden and accounts for 70% of all people living with HIV (PLHIV) 1. In this region, adolescent girls and young women (aged 15 to 24 years) are particularly vulnerable and, despite representing just 10% of the population, accounted for 25% of all new HIV infections in 2017. A recent report from a rural South African community illustrated the enormity of this problem where HIV prevalence was alarmingly high in women, reaching 40.1% in those 23 to 24 years and a staggering 71.7% in those aged 35 to 36 years 2. A complex interplay of biology, gender‐power disparities, socio‐economic and behavioural factors impacts vulnerability in women in Africa 3.

The female genital tract microbiome, which is the main site of infection for women, is a key, but lesser understood, biological factor affecting HIV susceptibility. In this review, we examine how disturbances in the female genital tract bacterial microbiome influence HIV acquisition and HIV prevention, describe the role of the rectal microbiome in HIV acquisition, outline advances in technologies to study the microbiome and postulate some future research directions in this field.

### 1.1 The human microbiome

For human hosts, the term “microbiome” includes all the genetic material of all the microbes (bacteria, fungi, protozoa and viruses) that exist in and on the human body. Trillions of microbial cells colonize the human skin and various mucosal niches 4, and are critically important for maintaining health. Disturbances in the microbiota, that is, all the microorganisms, in these various microbial niches have been linked to an array of adverse health outcomes, including altered gut microbiomes associated with inflammatory bowel disease 5, obesity 6, autoimmunity and heart disease 7, 8. Disturbances in the vaginal microbiome have been associated with reproductive complications (such as pelvic inflammatory disease, infertility, premature delivery, mid‐trimester pregnancy loss, amniotic fluid infection or low for gestational age infants, postpartum endometritis and gynaecologic postoperative infections 9. Transfer of gut bacteria can transmit phenotypes such as obesity and malnutrition to another animal both within and between species (for humans and mice) 10, 11, opening avenues for potential interventions. Transferring beneficial gut bacteria through faecal transplants has markedly improved the treatment of *Clostridium difficile* by improving the gut microbial environment 12.

In this review, we focus specifically on the female genital tract and rectal microbiomes and their association with HIV acquisition.

### 1.2 The female genital tract microbiome

The female genital tract microbiome comprises bacteria, protozoa, viruses and fungi inhabiting the human vagina, which may promote health (e.g. bacteria – Lactobacilli) or disease (e.g. bacteria – *Chlamydia trachomatis* and *Neisseria gonorrhoeae*; protozoa – *Trichomonas vaginalis*; viruses – Human papillomaviruses and Herpes simplex viruses; and fungi – *Candida* spp.). Studies 13, 14 examining whether there are differences between the cervical and vaginal microbiomes have revealed a high level of concordance in microbial diversity between the cervix and vagina. The composition of the female genital tract microbiome is unique to each woman and is probably established early on in life through exposure to key maternal microorganisms during birth 15, 16. Menarche and sexual debut also have an impact on the female genital tract microbiome, but their role has not been clearly characterized. While not fully understood, the microbial composition fluctuates naturally over time 16, particularly during hormonal shifts associated with puberty and menopause 17, 18 and through the menstrual cycle 17, 19.

In addition, several environmental factors such as hormonal contraceptives; sexual activity (including the number of partners and semen, as well as lubricants), hygiene practices, antibiotics and the composition of the gastrointestinal microbiota (transferred from the nearby rectum) can all influence the composition of the female genital tract microbiome 20, 21, 22, 23, 24, although it should be noted these data are not entirely consistent.

#### 1.2.1 Eubiotic microbiota

The female genital tract microbiome of healthy women asymptomatic for vaginal dysbiosis is usually dominated by one or two species of *Lactobacillus* (including *L. crispatus, L. iners, L. jensenii, L. mucosae and L. gasseri*). *Lactobacilli* spp. are thought to benefit the host by producing lactic acid (a potent, broad‐spectrum bactericide and virucide) and hydrogen peroxide, which lowers the vaginal pH (<4.5) and promotes the production of bacteriocins that reduce colonization by other common pathogenic microorganisms 25, 26, 27, including HIV and other sexually transmitted infections (STIs) 28, 29. However, not all *Lactobacillus* species contribute equally to the stability of the normal vaginal microflora. A study among pregnant women has shown that *L. gasseri* and/or *L. iners* may predispose the occurrence of abnormal vaginal microflora while presence of *L. crispatus* promotes the stability of the normal vaginal microflora 30.

#### 1.2.2 Dysbiotic microbiota

While it is generally thought that *Lactobacillus*‐dominant vaginal communities represent states of “health,” it is less clear whether all non‐*Lactobacillus* dominant communities are necessarily “unhealthy.” Several studies have highlighted racial and ethnic differences in healthy asymptomatic women 31, 32, 33. Generally, women of European and Asian ancestry are more likely to have a microbiota dominated by *Lactobacillus* while women of African or Hispanic descent are more likely to have non‐*Lactobacillus*‐dominated microbiota 34, 35, 36, 37. Up to 40% of apparently healthy women, with no clinical evidence of symptoms or discharge, have vaginal communities that lack appreciable numbers of *Lactobacillus* and instead have vaginal microbiomes that are dominated by a variety of other anaerobic bacteria 33, 34, 35, 38. This type of vaginal microbiome is common, especially in black and Hispanic women, and it is unclear whether this should be considered a “normal” or a dysbiotic/diseased state 38. The underlying factors determining this apparent tolerance of a non‐*Lactobacillus*‐dominated microbiota remain obscure. One hypothesis is that early‐life exposure to this type of vaginal microbiota could foster immunological tolerance 39. Other possibilities may relate to genetic variation between individuals and among ethnic populations, especially polymorphisms in cytokine, innate immunity and hormone‐response genes that could impact the threshold for immunological responses to bacteria in the vaginal tract 40, 41.

Furthermore, some anaerobes, like *Atopobium*,* Megasphaera* and *Leptotrichia* 35, 42, 43, are able to produce lactic acid and thus may be metabolically similar to species of *Lactobacillus* in their ability to lower vaginal pH 9, 16, 35, 38. However, it should be noted that these other lactic acid producers are frequently associated with bacterial vaginosis (BV) and high diversity vaginal bacterial communities, wherein *Atopobium*,* Megasphaera* and *Leptotrichia* are typically present at low relative abundance compared to *Lactobacillus* dominated vaginal bacterial communities, wherein *Lactobacillus* may account for up to 99% of the total bacterial relative abundance 44, 45, 46. Thus, the contribution of these relatively low‐abundance microbes to total lactic acid production in the vaginal tract may be minimal compared to vaginal communities that are dominated by *Lactobacillus*. In addition, their co‐occurrence with diverse anaerobes may counteract any pH lowering effects as some bacteria, including the common urogenital pathogen, *Neisseria gonorrhoeae*, have the capacity to take up lactic acid and utilize it as a carbon source, thus depleting it from their environments 47, and BV‐type microbial communities are associated with production of polyamines such as putrescine, cadaverine and trimethylamine, as well as ammonia which can counteract the pH lowering effects of lactic acid 48.

To simplify measurement of the female genital tract microbiome, several different algorithms have been applied to stratify women based on vaginal bacterial composition, by clustering them into various numbers of distinct Community State Types (CSTs, or vagitypes) 34, 38, 49, 50, 51, 52, 53, similar to enterotype clusters reported for the human gut microbiome 54. For example, the classification by Ravel and colleagues 38 proposed five CSTs where CST I, II, III and V are composed primarily of *Lactobacillus* species including *L. crispatus, L. gasseri, L. iners or L. jensenii* respectively while CST IV contains lower proportions of *Lactobacillus* species and a higher diversity of several other anaerobic organisms 38. CST IV was subsequently further divided into IVA (consisting primarily of *Bifidobacterium* spp.*, Dialister* spp.*, Streptococcus* spp. and Bacteroides spp.) and IVB (consisting of *Atopobium* spp.*, Gardnerella* spp.*, Mobiluncus* spp.*, Paryimonas* spp.*, Prevotella* spp.*, Megasphaera* spp. and several other anaerobic bacteria) 55. However, methodological differences, assessment of different populations of women and limitations of sequencing short regions of marker genes for taxonomic discrimination have contributed to the lack of a universal definition of CSTs. Despite their common usage and utility, it remains unclear whether this categorization adequately represents biologically and clinically relevant entities in the vaginal environment.

### 1.3 Dysbiosis of the vaginal microbiome, with a focus on bacterial vaginosis

An imbalance in the vaginal microbiota, characterized by a shift from a low‐diversity, *Lactobacillus*‐dominant state to a high‐diversity state in which the vagina is colonized by a wide range of strict and facultative anaerobes 40, 56, 57, 58, 59 and vaginal pH ≥4.5 57, 58, 60, is associated with a clinical condition referred to as bacterial vaginosis (BV). Although BV is not considered to be an infection (STI), it is known to be associated with condomless sexual contact 40, 61. Despite BV being common in reproductive‐age women, it remains an enigma in women's health. More than 50% of women with BV are asymptomatic 62, 63, highlighting the complexity of referring to BV as an illness. The complexity surrounding BV arises, in part, from a nebulous definition of the disorder and subjective diagnostic criteria 43.

Nugent scoring is most commonly used to diagnose BV in a research setting, based on the presence or absence of Lactobacilli rods by Gram staining 64. Alternatively, BV is diagnosed clinically based on the presence of three of the four Amsel criteria: (1) pH of vaginal fluid > 4.5, (2) presence of clue cells, (3) vaginal discharge and (4) malodour 62. BV‐associated symptoms and categories of vaginal dysbiosis (i.e. *Lactobacillus* dominant vs. non‐*Lactobacillus* dominant); however, may lack the requisite granularity to enhance our understanding of the complex aetiology of BV, discriminate important aspects of bacterial community structure and function, and discern host‐microbiome interactions that may serve to facilitate or inhibit HIV transmission.

Women with BV typically have a spectrum of anaerobes, including Gram‐positive anaerobic bacteria like *Gardnerella vaginalis*,* Atopobium vaginae*,* Eggerthella* spp., *Peptoniphilus* spp. and BV‐associated bacteria (BVAB1‐3, *Clostridia*‐like bacteria), anaerobic Gram‐negative bacteria like *Mobiluncus* spp., *Prevotella* spp., *Fusobacterium nucleatum* and *Megasphaera* type 1, and bacteria without cell walls, like *Mycoplasma hominis* and *Ureaplasma* spp 38, 45, 57, 61. Of these, biofilm‐producing *G. vaginalis* is thought to play a pivotal role in the initiation and persistence of BV 65, 66.

In one of the earliest studies seeking to understand the aetiology of BV, Criswell et al. 67. inoculated pure cultures of *G. vaginalis* into the vaginas of 13 healthy women. Only one of the 13 women developed BV. Separately, he inoculated 15 women with vaginal fluid from women with BV; and 11 of the 15 developed BV 61, 67. Subsequently, a wide range of host and environmental factors have been implicated in the aetiology of BV, including cigarette smoking 68, early age of sexual intercourse 69, oral sex 23, new or multiple sexual partners 21, menses and having intercourse while menstruating 19, and frequent douching (more than once a week) 70. However, several of these factors may be confounded, as many of the socio‐demographic factors that increase the risk of BV are more common in black women; or it may be that the symptoms of BV (such as malodour and discharge) cause women to douche rather than that douching has a direct effect on vaginal microbes 32, 71.

Sexual partners are thought to be a reservoir for BV‐associated bacteria 61. *G. vaginalis*,* A. vaginae*, BVAB1 and *Megasphaera* sp. type 1 have all been detected in the male partners of women with BV, with the coronal sulcus suggested to have the highest concentrations 72. In addition, semen was found to harbour high concentrations of bacteria, with some of the most abundant genera being *Prevotella, Streptococcus, Staphylococcus* as well as *Lactobacilli* 73. The penile skin and urethral microbiota of male partners of women with BV were more similar to the vaginal microbiota of their female partners than the vaginal microbiota of non‐partner women with BV 72. However, a recent meta‐analysis of seven placebo‐controlled trials, that included more than 1700 participants, showed that treating male partners with antibiotics does not significantly improve BV treatment outcomes or reduce recurrence in female partners 74. A limitation of these early trials were that test of cure was not conducted in the male partners, who were only treated with oral antibiotics, and the primary outcome of the trials was clinical cure (Amsel criteria) in women within one to two months of partner treatment. More recently, Plummer et al. 75 revisited this approach, using 16S rRNA gene sequencing and combined oral metronidazole and topical penile clindamycin treatment of male partners of women with BV, to show that a more homogeneous microbiota can be restored in women with this combined treatment approach, with a reduction in BV‐associated bacterial species. Furthermore, partners had an immediate effect on the penile cutaneous microbiota. Since male partner‐derived BV‐associated bacteria can colonize the lower FGT, it is probable that this microbiome sharing does modulate risk for HIV infection in women by causing BV, in addition to influencing topical PrEP efficacy.

A different school of thought is that the overgrowth of pathogens characteristic of BV has to be preceded by a major disturbance in the vaginal *Lactobacillus* population, such as specific targeting by bacteriophages 76, 77, 78. Bacteriophages targeting *Lactobacilli* have been isolated from various environmental sources, such as dairy products 79 and the human gastrointestinal tract 80. In addition, temperate phages are evidence in sequences from a wide range of human vaginal *Lactobacilli* spp. that are capable of infecting other *Lactobacilli* isolates from the same and different women 81, 82. In trying to understand major drivers of the shift from microbial health to dysbiosis in the vagina, significantly more work is required to better understand what BV is, how other constituents of the microbiome interact with *Lactobacilli,* and how to prevent and durably treat this common condition.

### 1.4 Dysbiosis of the female genital tract microbiome and the risk of HIV

Disturbances in the vaginal microbiota, associated with BV, increase genital inflammation and HIV target cell activation, likely through inflammatory cytokine induction 45, 83, 84. Raised pre‐infection genital concentrations of inflammatory cytokines (macrophage inflammatory protein (MIP)‐1α and MIP‐1β) were present in women who acquired HIV infection compared to women who remained uninfected 83. These inflammatory changes were associated with elevated levels of highly activated T cells in the genital mucosa 45, 84 and decreased efficacy of the antiretroviral drug tenofovir, which is a reverse transcriptase inhibitor that was assessed for its effectiveness in preventing HIV infections 85. In rhesus macaques, the establishment of a productive simian immunodeficiency virus (SIV) infection in the genital mucosa required local chemokines, which facilitated recruitment of CD4+ T cells to the site of infection, and enabled SIV expansion and establishment of infection 86. Li et al. 86 further showed that suppression of inflammation prior to SIV inoculation prevented SIV infection. In addition to recruiting target cells to the site of transmission, there is evidence showing that pro‐inflammatory cytokines facilitate HIV transmission by reducing the integrity of the epithelial barrier in the female genital tract and directly promoting HIV replication through NF‐kB activation 87, 88, 89.

While inflammatory cytokines are induced during BV, several chemokines are down‐regulated, including those associated with HIV risk (interleukin (IL)‐8, interferon‐γ inducible protein (IP)‐10 and monocyte chemotractant protein (MCP)‐1) 83. The reasons for suppressed chemokine expression in women who have BV remain unclear 40. Since BV is a complex polymicrobial condition, identifying precisely which microbes are associated with mucosal inflammation and barrier dysfunction is difficult. It is likely that this results from multiple pathogenic anaerobes 40.

Several recent studies have suggested that *G. vaginalis* and *P. bivia* contribute to both HIV risk and genital inflammation 45, 90, 91. Several virulence factors have been identified in both of these BV‐associated microbes. *G. vaginalis* produces several classes of cytolysin, including vaginolysin, which are capable of activating the protein kinase pathway in vaginal epithelial cells, inducing cell death 92. *Gardnerella* spp. also commonly produce sialidase, prolidase and putrescine, which degrade mucins, allow better microbial attachment and biofilm formation, and may contribute to epithelial sloughing during BV 93. Similarly, *Prevotella* spp., including *P. bivia*, produce collagenases, fibrinolysins, sialidases and prolidases 94. Metagenomic analysis of vaginal 16S sequences from women with BV suggested that lipopolysaccharide (LPS) from *P. bivia* was the strongest predictor of both genital inflammation and HIV risk in women 91. LPS signals through toll‐like receptor (TLR)‐4 and CD14, which are expressed by monocytes and macrophages, as well as epithelial cells in the cervix, vagina, fallopian tube and endometrium 95. TLR4 expression has been shown to decline progressively along the genital tract, being highest in the fallopian tubes and endometrium, and lowest towards the vaginal epithelium 96. Furthermore, some studies have suggested that the expression of TLR4 in the endometrium is hormonally regulated; being significantly higher during the secretory phase compared to other phases of the menstrual cycle 97. LPS binds to TLR‐4 and CD14 to activate the NF‐Kb cytokine pathway 95. *P. bivia* is a common constituent of BV, and known to produce high concentrations of LPS in vaginal washes 98. In an *in vitro* host cell cytotoxicity model using normal dopamine neuron cells, LPS from vaginal *P. bivia* induced cytotoxicity, although this needs to be confirmed in vaginal or cervical cells. Furthermore, in a study of inflammation caused by a periodontal *Prevotella* spp., *P. nigrescens* induced NF‐Kb inflammatory pathways involving nitric oxide in a murine macrophage model 99. It is therefore possible that this anaerobic pathobiont contributes substantially to genital inflammation, which may influence barrier disruption and increased HIV risk in women with BV.

### 1.5 Maintenance of vaginal health

Maintaining or restoring vaginal health is thought to play an important role in protecting women from reproductive complications and acquisition of STIs, including HIV and human papillomavirus (HPV) 45, 60, 70, 100, 101, 102, 103, 104, 105, 106, 107, 108, 109, 110, 111.

The most common treatment for BV includes either oral or topical metronidazole, although more than half of those receiving antibiotic treatment have recurrent BV within a year 112. More than 50 therapeutically bioactive compounds, including metronidazole, are known to undergo direct microbial modification in the gastrointestinal tract 113, 114, thereby altering their chemical structure and modifying their half‐life, bio‐availabilities and biological effects. Gut microbes predominantly use hydrolytic and/or reducing reactions to metabolize xenobiotics 115, and many of the enzymes associated with xenobiotic metabolism (including hydrolases, lyases, oxidoreductases and transferases) are common among gut microbial sequences 114. Although some of these modifications can be useful (conversion of inactive pro‐drugs into their active forms), many of these modifications lead to detoxification and/or inactivation of the compounds 113. Changes in metabolism of various drugs in germ‐free animals or following antibiotic treatment have confirmed the role of gut microbes in xenobiotic processing 115, 116.

Higher oestradiol levels have been shown to favour the dominance and stability of *Lactobacilli* spp. in the lower reproductive tract through the oestrogen‐mediated increase in the thickness and glycogen content of the vaginal epithelia, which peaks mid‐cycle 117, 118. Bacterial communities were shown to be more stable when oestradiol levels are at their highest, and the greatest change in the complexity of microbial communities was found at the onset of menstruation 19, 119, 120. A systematic review and meta‐analysis showed that some hormonal contraceptives reduce the risk of BV, through hormonal stabilization of vaginal microbial communities, arguing that a hormonal intervention could be useful in BV prevention 121, 122.

Pregnancy is an interesting endocrinological and immunological phase of life for women, where levels of both oestrogen and progesterone rise continually throughout pregnancy. Despite changing physiology, hormones and mucosal tolerance during pregnancy, several studies have shown that the vaginal community composition during gestation is relatively stable 16, 44, 123, with higher abundance of *Lactobacilli* spp. than non‐pregnant women, like *L. crisp*atus, *L. gasseri* and *L. jens*enii, and lower relative abundance of BV‐associated organisms, like *Prevotella, Sneathia, Gardnerella* and *Mobiluncus* 44. Romero et al. (2014) speculated that the higher vaginal community stability during pregnancy than non‐pregnancy may be a consequence of the physiological state of pregnancy 44. Similar to pregnancy, stability of vaginal microbial communities is highest around ovulation in non‐pregnant women 16, when oestrogen concentrations are high the vaginal epithelium mature, and epithelial glycogen stores as a carbon source for *Lactobacillus* spp. are higher 124. Pregnancy may therefore reflect an extended oestrogenic state, in the absence of menstruation, that favours Lactobacilli microbial stability.

Condom use can prevent BV. Women whose partners always used condoms were fivefold less likely to have persistent or recurrent BV 125. In contrast, women reporting less frequent condom use were less likely to have or sustain *Lactobacilli*‐dominant vaginal microbiota 126. Medical male circumcision protected women against BV. The female partners of men who participated in a randomized trial of circumcision to prevent HIV acquisition in Uganda were significantly less likely to develop BV than partners of uncircumcised men 61, 127. These findings suggest that male partners are a likely source of the organisms associated with BV, and transfer of these during condomless sex, particularly from male partners who have intact foreskins, results in displacement of commensal vaginal Lactobacilli.

*Lactobacilli*‐containing probiotics have had varying success in preventing BV recurrence, with a recent meta‐analysis including 1304 women from 12 RCTs, showing that probiotic supplementation significantly improved BV cure rates 128, although this was not significantly better than metronidazole treatment alone 129. However, long‐term colonization with the probiotic has proved difficult to achieve, although few products are explicitly for vaginal conditions and even fewer contain common vaginal *Lactobacilli* spp 130, 131, 132. It is likely that the same factors that caused disappearance of endogenous vaginal *Lactobacilli* in the first place may limit the colonizing success of introduced probiotic species.

### 1.6 The role of the rectal microbiome in HIV acquisition

In both men and women, receptive anal intercourse is associated with higher rates of HIV acquisition. The rectal immune environment is influenced by the gut microbiome, which a growing body of literature suggests is an important determinant of overall immune function 133, 134. The composition of the human gut and rectal microbiome change with age, influenced by a wide range of factors including diet, antibiotic use, gender and geographical location 135.

The gut has been a major focus of HIV research for over two decades, after initial reports of HIV‐related enteropathy. This was followed by demonstration of rapid gut CD4 depletion in both HIV‐infected humans and SIV‐infected non‐human primates in the days/weeks following infection 136, 137. Moreover, the resulting damage caused to the gut during this early phase of infection has been implicated as a driver of pathogenesis, specifically by causing translocation of microbial components into the blood stream and causing inflammation 138. Several studies have suggested that HIV alters the microbiome 139, 140, 141, 142. In addition to differences in species composition, altered bacterial metabolic activity has been noted in HIV infection 143, 144. However, recent studies have suggested that at least some of the differences between the gut microbiota of HIV‐positive and HIV‐negative individuals may be linked to sexual preference 145, 146, leaving an open question as to how HIV infection impacts the microbial composition of the gut, and in turn how this impacts its interaction with the host immune system (also greatly impacted by HIV). An even larger open question is the reverse relationship – whether the gut microbiota and associated immune environment of the rectum influence HIV susceptibility.

Comparatively, a greater role for commensal bacteria playing a role in HIV acquisition has been better described for the female genital tract 90, 111 than in the gut. While diversity in the vagina is likely a result of the loss of the physiologically critical *Lactobaccillus* spp., as discussed above, in the gut a diverse microflora is believed to be advantageous for improved nutrient absorption. While the extent to which the gut microbiome may be implicated in rectal HIV acquisition is unknown, the gut microbiome plays a critical regulatory role in defining the rectal immune environment by signalling through pattern recognition receptors 147, setting up what has been referred to as a “mucosal firewall” and controlling host‐advantageous communication with the external environment. The gut microbiome is also associated with mucosal Th17 and T*reg* frequencies 148, cell subsets that play important roles in HIV transmission 149, 150.

The role of rectal microbiota in HIV acquisition has been explored in recent animal and human studies. A study in non‐human primates demonstrated that differences in SHIV susceptibility was due primarily to gut microbial differences 151, as the more susceptible group of animals had higher levels of immune activation that were linked to lower *Bacteroides* and Firmicutes, and higher proportions of *Prevotella* spp. In a human study, condomless rectal sex was found to have a substantial inflammatory effect on the rectal immune environment, including on composition of the microbiota inflammation‐associated bacteria from the *Prevotellaceae* family 146. This microbial profile was also associated with increases in Th17 cells, pro‐inflammatory cytokines, and a gene expression signature of wound injury. These findings echo findings of the important inflammatory role for *Prevotella* in the female genital tract, suggesting that this bacterial species in the microbiome of both compartments may be contributing to HIV acquisition.

### 1.7 The female genital tract microbiome and its impact on antiretroviral drug concentrations

The composition of the vaginal microbiome has been shown to have a direct impact on the efficacy of some antiretroviral drugs that are used as pre‐exposure prophylaxis (PrEP) for HIV prevention, particularly those administered intravaginally. In one study, the effectiveness of topical tenofovir gel against HIV was diminished substantially in women with BV‐associated bacteria compared to women with a *Lactobacillus*‐dominated microbiota 90. In this study, tenofovir concentrations were significantly depleted *in vitro* (by >50%) within four hours in cultures with *G. vaginalis* (the bacterial isolates that were most common in women with non‐*Lactobacillus* dominated microbiota) compared to only marginal changes in cultures with *Lactobacillus species*. In addition, the tenofovir metabolite, adenine, increased progressively in *Gardnerella* cultures as tenofovir concentrations declined, indicating that this BV‐associated organism was metabolizing tenofovir 90. The metabolism of tenofovir by *G. vaginalis* has also been shown in a recent study 152 that found that tenofovir concentrations were lower in both cervicovaginal fluid and plasma of women with BV‐associated *G. vaginalis* and *A. vaginae* after applying tenofovir gel or tenofovir film for a week. Dapivirine, the antiretroviral being used in intravaginal rings for HIV prevention, has also been shown to be impacted by BV‐associated bacteria, although the mechanism differs from tenofovir 153. Unlike tenofovir, dapivirine does not require intracellular modifications and its pharmacokinetics are therefore not disrupted by BV organisms. Instead, *Gardnerella* spp. appear to bind dapivirine irreversibly, thereby reducing drug concentrations 153, 154. Recent studies have found that tenofovir alefenamide, a pro‐drug of tenofovir, was not metabolized by BV‐associated bacteria suggesting it may be a more appropriate antiretroviral drug for PrEP in women with BV 153, 154. The composition of the vaginal microbiome; however, does not appear to impact on the effectiveness of oral tenofovir‐containing PrEP 155.

### 1.8 Changing technologies to study the structure and function of the genital microbiome

The application of –omic technologies to assess the vaginal and rectal microbiome and host responses to dysbiotic states have the potential to provide a greater systems‐level understanding of states of health and disease that influence the risk for STIs such as HIV, particularly when integrated with measures of host physiology, genetics and environmental and behavioural factors. Such a systems approach may be particularly important in the context of HIV susceptibility wherein microbial and host factors and their inextricable and complex interactions at mucosal sites may promote conditions that either increase or reduce the risk of HIV acquisition. As the application of newly evolving technologies expands the ability to study microbiome‐host interactions in the vaginal ecosystem, these tools can be applied more broadly to other mucosal ecosystems, including the rectal mucosal environment.

#### 1.8.1 Amplicon‐based sequencing approaches

The advent of cultivation‐independent, next‐generation sequencing has dramatically advanced our knowledge of the diversity and complexity of the human microbiome. To date most studies of the vaginal microbiome have employed high throughput sequencing targeting bacterial marker genes, such as the 16S rRNA or chaperonin‐60 (cpn60) genes 25, 156, 157, 158. In addition to providing more comprehensive surveys of vaginal microbial communities, 16S rRNA gene sequencing has led to the identification of previously unrecognized BV‐associated bacteria in the order Clostridiales; BVAB1, BVAB2 and BVAB3 (now isolated and classified as *Mageeibacillus indolicus*) 159, 160, 161, and cpn60 sequencing has been utilized to resolve different *G. vaginalis* subgroups which may have variable clinical and pathological relevance for BV 162, 163, 164, 165, 166.

Although compositional analyses using marker gene sequencing have led to important insights into the complexity of the vaginal microbiota as it relates to BV, vaginal inflammation and HIV acquisition 49, 110, 111, 167, it has several important limitations that need to be considered, such as the inability to provide fine scale taxonomic resolution (species‐ and strain‐level), that these are subject to primer biases, and that they cannot provide exact information about the metabolic functional capacity of bacterial communities.

#### 1.8.2 Shotgun metagenomic sequencing

Shotgun metagenomic sequencing (SMS), while more costly and computationally demanding, can overcome many of the limitations of amplicon‐based sequencing by improving taxonomic classification. SMS can also identify other important components of the vaginal microbiome (such as viruses, fungi, archaea and protozoa) and thereby shed light on the broader relationships with the vaginal microbiome that cannot be appreciated using bacterial amplicon‐based approaches alone. Despite these advantages, only a few published vaginal SMS datasets are currently available; one was derived from a subset of 113 U.S. adult females as part of the Human Microbiome Project (HMP) and another from a recent longitudinal study of 10 pregnant women in the U.S 52, 168. Analyses of HMP vaginal SMS data revealed an association between vaginal pH and metabolic diversity. However, this U.S. cohort may be inadequate to assess states of vaginal health and disease as very few women had vaginal pH >4.5 169. Anahtar et al. applied SMS to evaluate functional differences in the female genital tract microbiome of 12 black South African women from the FRESH cohort (six women with high vs. six with low genital inflammation) and found that the pathway for lipopolysaccharide biosynthesis was enriched in the vaginal metagenomes of women with high inflammation 45. Additionally, in 180 women from the FRESH cohort, Gosmann et al. assessed the female genital tract virome using SMS with a virus enrichment protocol, identifying alphapapillomaviruses, *Anelloviridae* and *Caudovirales* as predominant viral taxa. However, no relationship between the virome and bacterial communities based on amplicon‐sequencing were identified 111. Thus, there is a paucity of SMS datasets for the vaginal microbiome and a need for and greater appreciation of differences between women from different geographic populations. Assessment of vaginal microbial composition based on SMS is superior to amplicon sequencing in that it can provide species and even strain level resolution of bacterial taxa using marker gene approaches, such as MetaPhlAn2 52, alignment of raw sequences to whole genome databases 170, or genome assembly‐based approaches 171, 172. However, such analyses are limited to bacterial taxa and strains for which full bacterial genomes have been assembled and deposited in databases. Thus, genomes of certain important vaginal bacterial species and strains that may have clinical relevance are missing from databases for MetaPhlAn2 and whole genome databases (i.e. *Sneathia* spp., BVAB1, BVAB2, and likely others), and such omissions can have dramatic effects in skewing abundance profiles and misclassifying samples to CSTs, especially in samples where such bacterial taxa may be dominant. In this regard, whole‐genome sequencing and comparative genomics of vaginal bacterial isolates could advance the accurate assessment of the vaginal microbial community structure and function, as has been undertaken for select vaginal isolates 66, 173, 174, 175, 176. One of the greatest strengths of SMS is the gene‐ and function‐level information it provides on the microbiome. The functional metabolic capacity of microbial communities could help to provide a clearer picture of vaginal health. Given that some BV‐associated taxa are also capable of lactic acid production, microbial community function could be a greater predictor of vaginal health that is dependent on context (i.e. HIV risk or susceptibility to other STIs as opposed to BV symptoms). SMS datasets in large cohorts would help to distinguish such context‐specific definitions of health and disease.

#### 1.8.3 Metatranscriptomics and metaproteomics

Complementary ‐omic technologies aimed at evaluating the functional capacity of the microbiome, including metatranscriptomics and metaproteomics, offer further opportunities to expand our understanding of gene transcriptional activity and protein expression respectively in complex microbial communities inhabiting the human body. To date, metatranscriptomics with RNA‐seq has only been exploited in a handful of small studies of the vaginal microbiome 58, 177, 178. Vaginal bacterial metatranscriptome comparison between two women with BV and two healthy individuals indicated that in BV, *L. iners* increases transcription of cholesterol‐dependent cytolysin, genes involved in glycerol metabolism, and CRISPR‐related genes 58. Comparing the vaginal bacterial metatranscriptome in BV between metronidazole treatment responders and non‐responders, found that increased transcription of CRISPR‐Cas genes in *G. vaginalis* in non‐responders may mitigate treatment resistance 178. Metatranscriptomic analyses of cultured vaginal bacteria under different growth conditions may also provide important information. *In vitro* growth of *G. vaginalis* isolates in planktonic culture compared to biofilm growth has revealed extensive differences in the metatranscriptome that may be important for biofilm persistence and virulence 179. The emerging field of metaproteomics, studying bacterial proteins by mass spectrometry, is further shedding light on functionality of the vaginal microbiome and its relationship with BV, as well as antiretroviral drug efficacy, and has recently been extensively reviewed 180. Metaproteomics can provide important information on vaginal bacterial community structure, diversity and function and can further be applied to cultured isolates to better understand the protein repertoire expressed by individual vaginal bacteria. More recently, a “cell shaving proteomic” approach was used to identify 261 cell surface‐associated proteins of *G. vaginalis* in culture 181. This technology has several advantages as the metaproteome reflects the actual protein output, which may diverge from measures of bacterial community structure defined by amplicon sequencing, gene content (SMS) or metatranscriptomes 180, 182, 183.

#### 1.8.4 Metabolomics

The human microbiome, including the vaginal ecosystem, executes a wide range of metabolic activities, producing a plethora of metabolites in the process, which may underlie important microbe‐host driven mechanisms distinguishing phenotypic traits. Advancements in both targeted and comprehensive untargeted metabolomics have already provided evidence for divergent metabolic activity associated with vaginal dysbiotic states, including abnormalities in sugar compound, short chain fatty acid, lipid, biogenic amine and amino acid metabolism 48, 184, 185, 186, 187, 188, 189, 190. However, little is known about vaginal metabolic profile disturbances in the context of STIs 191 or metabolite biomarkers that may be associated with infection risk 192, 193. Studies assessing vaginal metabolic profiles in the context of BV treatment (such as rifaximin) efficacy, can inform the development of effective clinical treatments 194. Greater appreciation of the impacts of individual bacterial metabolites on vaginal physiology could provide important mechanistic insights into states of vaginal health and disease. It should further be noted that the expansive metabolic capacity of the microbiome can impact drug efficacy and safety, as some bacteria of the microbiome can directly metabolize xenobiotic 195, 196, 197 or impact drug responses through immune‐mediated mechanisms, as suggested for immune checkpoint inhibitors used in cancer treatment 197, 198, 199, 200.

### 1.9 Suggestions for future research

With the wide range of available –omic technologies, the microbiome research field stands poised to address many of the questions that have led to an inadequate definition of vaginal health and the factors mediating STI outcomes. However, a better functional definition of BV and vaginal states that influence STI risk, in addition to microbial factors, will also require substantial effort to understand human host intrinsic (hormonal fluctuations, genetic polymorphisms, inflammatory responses), behavioural (sexual activity, contraception usage, vaginal cleansing practices, stress, physical activity, smoking, diet) and sociodemographic (age, ethnicity, geographic location) factors that may influence interactions at the host‐microbe interface. Given the temporal instability of the vaginal microbiome 16, 201, longitudinal, rather than cross‐sectional, study designs will further help to elucidate factors mediating transitions between healthy and unhealthy states. Finally, the application of multi‐omic approaches to study the microbiome and the host, leading to the acquisition of detailed study metadata will require novel approaches for integrating and interpreting such high dimensional datasets. Network modelling and machine learning approaches that can contend with such heterogeneous, complex datasets are being used increasingly in biology 202, 203, 204, 205. Some such analytical approaches, like MIMOSA, which facilitates integration of bacterial community data with metabolomic data, have been developed specifically with a focus on improving our understanding of the microbiome 190. Undoubtedly, additional bioinformatic tools are already in development that will further our understanding of the complex interactions between the microbiome and human factors in multiplex, fluctuating biological environments such as the vaginal tract.

## 2 Conclusions

In conclusion, the vaginal microbiome is a complex environment that is continually shifting. Imbalances in the vaginal microbiota are associated with a range of adverse reproductive health outcomes, including increasing the risk of genital inflammation and HIV acquisition. While there does not appear to be any impact of BV on oral PrEP effectiveness, the finding that BV‐associated organisms actively undermine HIV prevention modalities by degrading antiretroviral drugs, there is an urgent need to consider a more generalized role of these microbes in modifying the effect of biomedical HIV prevention tools, and whether the effect extends to long‐acting or systemic PREP strategies. However, maintaining vaginal health with antibiotics and probiotics has proved challenging. A better understanding of the composition, structure and function of the microbiome through advances in sequencing technologies may enable us to develop new strategies to make the vaginal microbiome healthy and prevent the occurrence of BV and reduce the risk of HIV acquisition.

## Competing interests

The authors declare no conflicts of interest.

## Authors’ contributions

The authors contributed equally to this review.
